# The Ties That Bind: How Online and Offline Interactions Affect Social Support and Quality of Life for Older Adults

**DOI:** 10.1093/geroni/igab046.1166

**Published:** 2021-12-17

**Authors:** Shelia Cotten

## Abstract

Though a digital divide still exists, older adults are increasingly using a range of information and communication technologies (ICTs) – smartphones, apps, tablets, and computers – to communicate and engage with social ties. This symposium focuses on modalities of interaction – whether online or offline – that older adults use to interact with social ties. The research projects detailed examine the frequency of different interaction modalities, as well as impacts of these interaction modalities on older adults’ perceptions of social support and quality of life. Kadylak and colleagues focus on social robots and how older adults may engage with this evolving technology to improve social engagement and aging in place. Kim and Fingerman investigate whether daily social media use is associated with same-day negative or positive mood in later life. Xie and colleagues examine older adults’ patterns of both online and offline social interaction during COVID-19, and how older adults perceive these interactions. Schuster and Cotten, using a national sample of individuals aged 65 and older, examine whether social media use may be related to a range of quality of life indicators. Each of these studies provides additional insights into the ways through which older adults interact and communicate with social ties, and potential impacts of the different ways through which they interact, which may provide insights into groups seeking to increase social engagement among older adults in general and during times when social isolation may be exacerbated due to societal stressors, such as pandemics.

